# Apalutamide for prostate cancer: Multicentre and multidisciplinary real‐world study of 227 patients

**DOI:** 10.1002/cam4.6769

**Published:** 2023-12-08

**Authors:** Julián Córdoba Sánchez, Natalia Picola, Alejo Rodriguez‐Vida, Marc Costa, David Marmolejo Castañeda, Meritxell Pérez Márquez, Jesús Muñoz Rodriguez, J. M. Gaya, Alejandra Bravo, Oscar Buisan, Pol Servian, Jose Francisco Suarez, Mireia Musquera Felip, Maria Jose Ribal Caparrós, Antonio Alcaraz Asensio, Antoni Vilaseca

**Affiliations:** ^1^ Uro‐Oncology Unit Hospital Clínic Barcelona Spain; ^2^ Urology Department Hospital Belltvige Barcelona Spain; ^3^ Medical Oncology Department Hospital del Mar Barcelona Spain; ^4^ Urology Department Hospital Val D'Hebron Barcelona Spain; ^5^ Medical Oncology Department Hospital Vall D'Hebron Barcelona Spain; ^6^ Urology Department Consorci Sanitari de Terrassa Terrassa; ^7^ Urology Department Hospital Parc Taulí Sabadell; ^8^ Urology Department Fundació Puigvert Barcelona Spain; ^9^ Urology Department Hospital Universitari Germans Trias i Pujol Badalona Spain

**Keywords:** apalutamide, high‐risk non‐metastatic castration‐resistant prostate cancer, metastatic hormone‐sensitive prostate cancer, prostate cancer, real‐world

## Abstract

**Objective:**

To evaluate the efficacy and safety of apalutamide prostate cancer compared to the pivotal trials patients and to identify the first subsequent therapy in a real‐world setting.

**Methods:**

The study is prospective and observational based on real‐world evidence, performed by different medical disciplines and eight academics centres around Barcelona, Spain. It included all patients with metastatic hormone‐sensitive prostate cancer (mHSPC) and high‐risk non‐metastatic castration‐resistant prostate cancer (nmCRPC) treated with apalutamide from June 2018 to December 2022.

**Results:**

Of 227 patients treated with apalutamide, 10% had ECOG‐PS 2, and 41% were diagnosed with new‐generation imaging. In the mHSPC group (209 patients), 75 years was the median age, 53% had synchronous metastases, and 22% were M1a. In the nmCRPC (18 patients), 82 years was the median age, and 81% ≤6 months had PSA doubling time. Patients achieved PSA90 in 92% of mHSPC and 50% of nmCRPC and PSA ≤0.2 in 71% of mHSPC and 39% of nmCRPC. Treatment‐related adverse events occurred in 40.1% of mHSPC and 44.4% of nmCRPC. After discontinuation of apalutamide due to disease progression, 54.5% in mHSPC and 75% in nmCRPC started chemotherapy, while after discontinuation because of adverse events, 73.3% in mHSPC and 100% in nmCRPC continued with other hormonal‐therapies.

**Conclusions:**

The efficacy and safety of apalutamide were similar to that described in the pivotal trials, despite including an older and more comorbid population. Usually, subsequent therapies after apalutamide differed depending on the reason for discontinuation: by disease progression started chemotherapy and by adverse events hormonal sequencing.

## Graphical abstract

This study is prospective and observational based on real‐world evidence, performed by different medical disciplines and eight academic centres around Barcelona, Spain. It included all patients with metastatic hormone‐sensitive prostate cancer (mHSPC) and high‐risk non‐metastatic castration‐resistant prostate cancer (nmCRPC) treated with apalutamide. Of 227 patients treated with apalutamide, 10% had ECOG‐PS 2, and 41% were diagnosed with new‐generation imaging. In the mHSPC group (209 patients), 75 years was the median age, 53% had synchronous metastases, and 22% were M1a. In the nmCRPC (18 patients), 82 years was the median age, and 81% of ≤6 months had PSA doubling time. Patients achieved PSA90 in 92% of mHSPC and 50% of nmCRPC and PSA ≤0.2 in 71% of mHSPC and 39% of nmCRPC. In conclusion, the efficacy and safety of apalutamide were similar to that described in the pivotal trials, despite including an older and more comorbid population. Usually, subsequent therapies after apalutamide differed depending on the reason for discontinuation: by disease progression started chemotherapy and by adverse events hormonal sequencing.

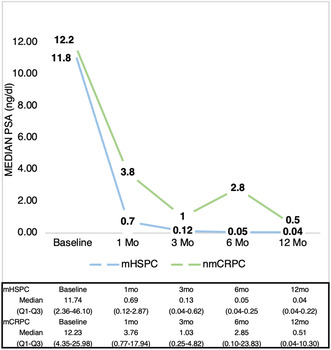

## 1 INTRODUCTION

Prostate cancer (PC) is the second most frequent cancer in men worldwide, with an estimated incidence of 1.4 million and a mortality of 375,000 patients in 2020.^1^ In Catalunya, 5227 new cases and 910 deaths were registered in 2020.^2^ Apalutamide is an oral non‐steroidal anti‐androgen that binds directly to the ligand‐binding domain of the androgen receptor (AR) and prevents AR translocation, DNA binding, and AR‐mediated transcription.^3^ It has now been shown that direct inhibition of the AR with ADT plus treatment intensification with apalutamide can provide a more complete blockade of signalling and have better oncologic outcomes in patients with metastatic hormone‐sensitive prostate cancer (mHSPC) and high‐risk non‐metastatic castration‐resistant prostate cancer (nmCRPC).^4^, ^5^

In 2018, the SPARTAN phase 3 trial showed that apalutamide increased metastasis‐free survival and other oncologic outcomes including overall survival (OS) in high‐risk nmCRPC patients compared to placebo. Participants in that pivotal trial were diagnosed with conventional imaging (CT scan and bone scan).^4^ However, the use of next‐generation imaging (NGI) has been shown to be able to identify metastasis in 58% of patients previously diagnosed with non‐metastasic high‐risk disease by conventional imaging.^6^

In 2019, the TITAN phase 3 trial reported increased radiographic progression‐free survival and OS with an acceptable safety profile in patients with mHSPC treated with apalutamide compared to placebo. Similarly, the population included in this pivotal trial were only diagnosed with conventional imaging and excluded patients with only nodal metastases (M1a) or patients with significant cardiovascular comorbidities.^5^

In 2021, the final analysis of SPARTAN and TITAN trials confirmed and broadened the superior oncologic benefits of apalutamide group compared to the placebo group.^7^, ^8^ Furthermore, a post‐hoc analysis of SPARTAN trial showed that PSA response with apalutamide is associated with short‐ and long‐term oncological benefits, supporting the use of prostate‐specific antigen (PSA) monitoring in routine clinical practice.^9^ Similarly, a post‐hoc analysis from the TITAN trial reported an association between PSA response and better oncologic outcomes, confirming its role as a surrogate marker of benefit and survival.^10^

Although randomized clinical trials (RCTs) achieve the highest level of evidence due to its great power to infer causality and prove the efficacy of a medical intervention in patients^11^ they have some limitations. One of the most common limitations in prostate cancer studies is the underrepresentation of some group populations, such as elderly or comorbid patients, which are very common in this disease. Therefore, real‐world analysis generates important data that complement and/or amplify the information obtained from RCTs.^12^, ^13^, ^14^, ^15^ The aim of our study is to evaluate the efficacy and safety of apalutamide among patients treated in a real‐world setting with broader inclusion criteria compared to the pivotal trials population and to identify the first subsequent therapy, in order to gain insight into the most common treatment sequences in daily practice.

## 2 METHODS

### 2.1 Study design

The study is a prospective observational cohort based on real‐world evidence. Subsequently, every patient who started apalutamide was added and followed synchronously from January 2021 until December 2022. It was performed by different medical disciplines in eight academic centres around Barcelona, Spain. The ethics and clinical research committee of the Hospital Clínic de Barcelona approved this study (HCB/2019/0919), and the institutional review board of each centre validated it. The patient data collection was conducted in compliance with the Helsinki Declaration and the good clinical practices established in the international regulations on medical ethics.

### 2.2 Study patients

This study included all patients with mHSPC or high‐risk nmCRPC treated with apalutamide from June 2018 to December 2022, diagnosed by either standard or new‐generation imaging. Patients were classified into two groups according to disease stage in mHSPC or nmCRPC.

### 2.3 Study variables

Patients' characteristics variables included age, follow‐up time, ECOG PS classification, initial diagnostic imaging classification, Gleason and ISUP grades, classification of mHSPC by volume and risk of disease and moment of appearance of metastases. In nmCRPC, the classification of risk was done according to the PSA doubling time (PSADT). The imaging method for the diagnosis of the disease was also reviewed. Data on pharmacological interactions (PI) with apalutamide were collected.

Efficacy variables included PSA levels at baseline, at 1, 3, 6 and 12 months of treatment, nadir PSA (which was defined as the lowest PSA level obtained by patients with a minimum of two tests and one of them at 6 months or with only one test at any time with an undetectable PSA level), time to nadir PSA and discontinuation of treatment because of disease progression, time to discontinuation and subsequent treatment. PSA50, PSA90 and PSA≤0.2 and time to response were calculated.

Safety variables were the type of adverse events (AE) and their respective grades, time to onset of the AE, time to resolution of the AE, need for dose reduction, interruption or discontinuation of apalutamide due to AE and their respective times. Furthermore, the dates and reason for starting a subsequent line of therapy after the apalutamide discontinuation was collected.

### 2.4 Statistical analysis

This study was predominantly descriptive. The types of metrics used for the pivotal trials were estimated. The number of valid data for each variable is described in Tables S1–S3. The data analysis was estimated using JMP Trial Software, version 17.

## 3 RESULTS

### 3.1 Patients and efficacy

A total of 227 patients were treated with apalutamide between June 2018 and December 2022 among these eight medical centres. All patients started therapy at a full dose of 240 mg daily. The observation time was of 24 months from 1 January 2021 to 31 December 2022. The median duration of apalutamide was 9.3 months in the mHSPC group and 19.8 months in the nmCRPC group. Patient and disease characteristics at baseline and oncological outcomes are described in Table 1, together with the results of the respective pivotal trials population.^4^, ^5^, ^9^, ^10^

**TABLE 1:** Characteristics of the patients and oncological outcomes.

	mHSPC	TITAN	nmCRPC	SPARTAN
Total of patients, *n*	209	525	18	806
Median follow‐up, month	12	22.7	21	20.3
Median age	75	69	82	74
Range	50–95	45–94	57–97	52–97
ECOG PS (%)				
0	58	62.5	73	77.3
1	32	37.5	9	22.7
2	10	0	18	0
Diagnostic technique (%)				
Conventional Imaging	58	100	72	100
New Generation Imaging	42	0	28	0
Gleason score (%)				
<7	0.5	7.8	6	19.4
7	32	25.3	34	37.1
>7	68	66.9	60	43.5
Metastatic disease, (%)				
Synchronous	53	78.3	‐	‐
Metachronous	47	16.2	‐	‐
Metastatic stage, (%)				
M1a	22	0	‐	‐
M1b–c	78	100	‐	‐
Disease volume, (%)				
Low	65	38.1	‐	‐
High	35	61.9	‐	‐
Disease risk (%)				
Low	61	44.9	‐	‐
High	39	55.1	‐	‐
PSA doubling time				
Median—month	‐	‐	5	4.4
≤6 months (%)	‐	‐	81	71.5
>6 months (%)	‐	‐	19	28.5
PSA baseline (ng/mL). median	11.74	5.97	12.2	7.78
(Q1–Q3)	(2.36–46.10)	(1.10–26.03)	(4.35–25.98)	(3.8–17.0)
Patients achieving PSA response in any time (%)				
Any decrease	100	‐	100	‐
PSA50	98	90	89	90
PSA90	92	73	50	62
PSA ≤ 0.2	71	68	39	38
Time to achieve PSA response—month				
Any decrease, median	1	‐	1	‐
(Q1–Q3)	(1.0–1.0)	‐	(1.0–1.0)	‐
PSA50, median	1	1	1	1
(Q1–Q3)	(1.0–1.0)	(1.0–1.0)	(1.0–1.5)	(1.0–1.0)
PSA90, median	1	1.9	1	1.9
(Q1–Q3)	(1.0–3.0)	(1.0–2.8)	(1.0–3.0)	(1.0–3.1)
PSA ≤ 0.2, median	3	1.9	3	2.8
(Q1–Q3)	(1.0–3.0)	(1.0–3.5)	(1.0–4.5)	(1.9–4.6)
PSA nadir (ng/mL), median	0.04	0.02	0.5	0.38
(Q1–Q3)	(0.04–0.15)	(0.02–0.32)	(0.1–11.8)	(0.1–1.6)
Time to PSA nadir, median—month	3	5.6	6	7.4
(Q1–Q3)	(3.0–6.0)	(3.7–12.9)	(3.0–12.0)	(4.5–11.9)

*Note*: The numerators and denominators per variable adjusted according to the amount of valid data in this study are described in Table S1.

**FIGURE 1:**
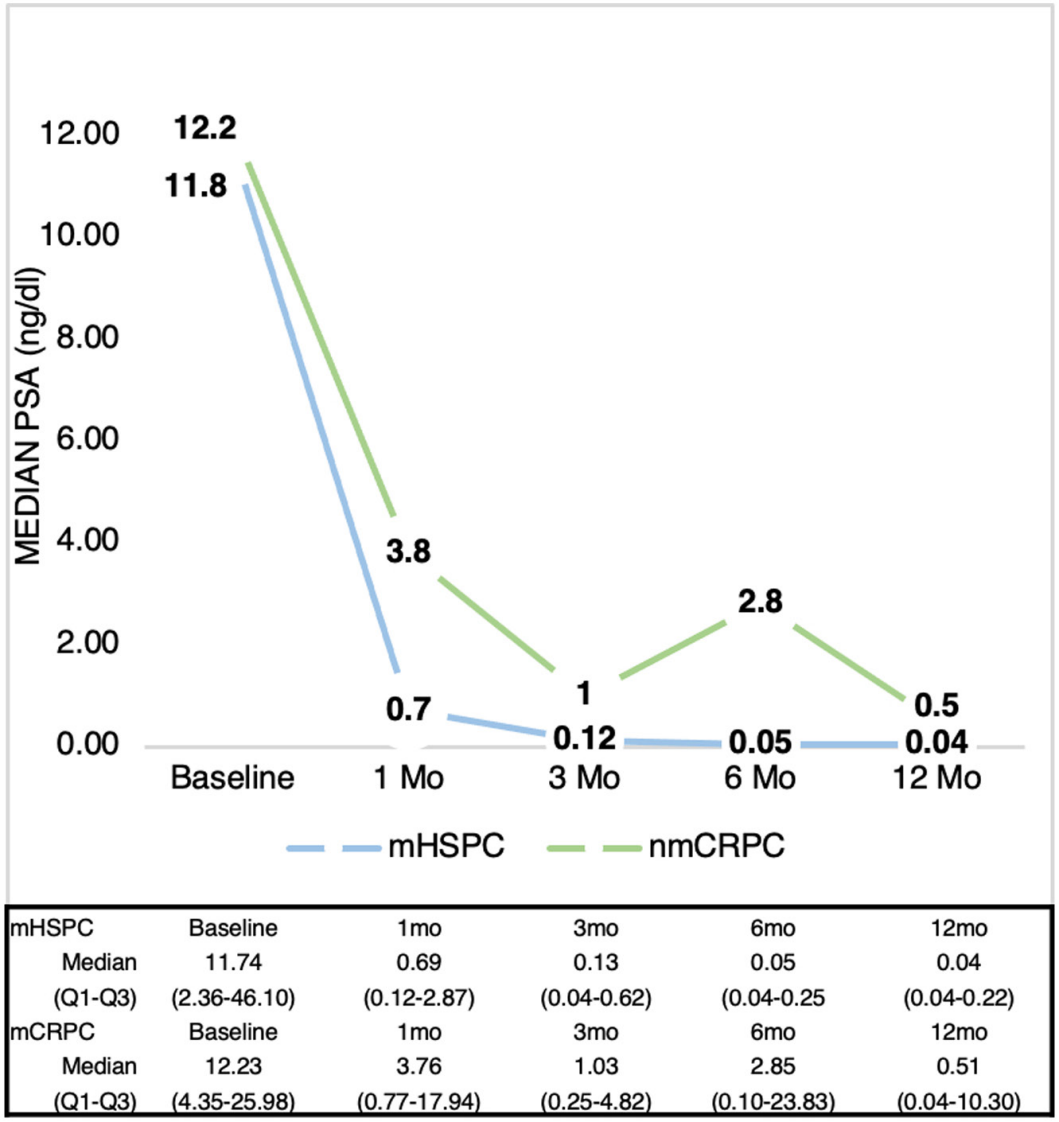
PSA evolution represents a rapid, profound and durability reduction of PSA in most patients.

**FIGURE 2:**
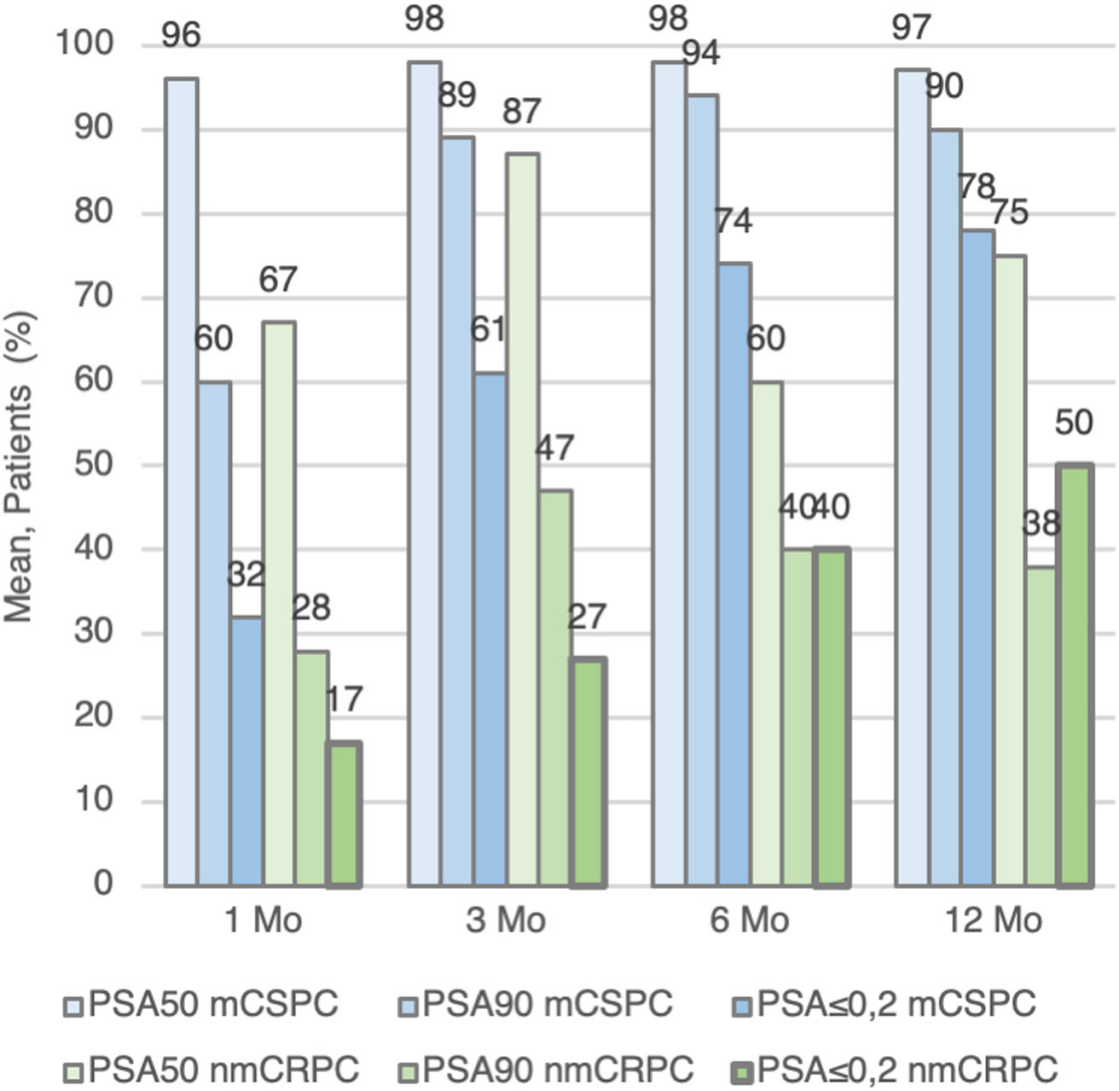
Kinetics of PSA describes the variations of proportions of patients who achieved decreased PSA of 50%, 90% or lower than 0.2 ng/dL for each month analysed.

With respect to PSA kinetics, a satisfactory response was observed at all time points, except at the 6‐month measurement in patients with nmCRPC, due to disease progression. Figure 1 represents the median PSA evolution of the whole cohort. Figure 2 represents the PSA kinetics (PSA50, PSA90 and PSA ≤0.2) of our cohort.

### 3.2 Safety

Table 2 presents a summary of AE and the most frequent types of AE observed in this study together with the AE described in the pivotal population.^4^, ^5^ In addition, 8.1% of patients in the mHSPC group and 16.6% of patients in the nmCRPC group presented one or more PI with apalutamide. Two patients in the mHSPC group presented serious adverse events because of PI with acenocumarol (stroke) and larcosamide (seizures), requiring hospitalizations and discontinuation of apalutamide.

**TABLE 2:** Adverse events (AE).

	mHSPC	TITAN	nmCRPC	SPARTAN
Patients with AE, (%)				
Any AE and all grades	40.1	96.8	44.4	96.5
Any AE and grade ≥3	11.5	42.2	16.6	45.1
Any AE leading to death	0	1.9	0	1.2
Time to AE, days	84	‐	79	‐
Median (Q1–Q3)	(35–161)	‐	(77–119)	‐
Time to AE resolution, days	70	‐	42.5	‐
Median (Q1–Q3)	(35–112)	‐	(20–77)	‐
Most frequent adverse events				
Rash				
All grades, (%)	15.8	27.1	16.7	23.8
Grade ≥ 3, (%)	5.7	6.3	11.1	5.2
Asthenia or Fatigue				
All grades, (%)	18.2	26.8	0	30.4
Grade ≥ 3, (%)	2.9	3.2	0	0.9
Arthralgia				
All grades, (%)	2.4	17.4	5.6	15.9
Grade ≥ 3, (%)	1	0.4	0	0
Hypertension				
All grades, (%)	2.4	17.7	5.6	24.8
Grade ≥ 3, (%)	1.4	8.4	0	14.3
Weight alteration				
All grades, (%)	1	10.3	0	16.1
Grade ≥ 3, (%)	0.5	1.1	0	1.1
Diarrhea or constipation				
All grades, (%)	1.4	9	11.1	20.3
Grade ≥ 3, (%)	0	0	0	1
Hypothyroidism				
All grades, (%)	1.4	6.5	5.6	8.1
Grade ≥ 3, (%)	0	0	0	0
Blood alkaline phosphatase increased				
All grades, (%)	1	3.1	0	0.2
Grade ≥ 3, (%)	0	0.4	0	0

*Note*: The numerators and denominators per variable adjusted according to the amount of valid data in this study are described in Table S2.

### 3.3 Second‐line therapy

After discontinuation of apalutamide due to disease progression, 54.5% of patients with mHSPC and 75% with nmCRPC started chemotherapy. Conversely, following discontinuation of apalutamide due to AE, 73.3% of patients with mHSPC and 100% with nmCRPC continued with other hormonal agents. Table 3 describes the types of therapy modification because of disease progression or AE, times to modification and subsequent therapy in our cohort together with data from pivotal studies.^4^, ^5^

**TABLE 3:** Modification of treatment and subsequent therapy.

	mHSPC	TITAN	nmCRPC	SPARTAN
Patients who discontinued apalutamide for any reason (%)	13.9	32.4	44.4	39.1
Patients who discontinued apalutamide because of disease progression (%)	5.7	18.9	33.3	19.3
Time to discontinue apalutamide because of disease progression. Median (IQR)—month	12	‐	19.5	‐
	(9–13)	‐	(12.5–35.5)	‐
Patients with AE leading to discontinuation of apalutamide (%)	7.2	8	11.1	10.6
Time to discontinuation of apalutamide because AE. Median (IQR)—month	2	‐	4.5	‐
	(1.5–4)	‐	(3.75–5.25)	^a^
Deaths from any cause (%)	1	15.8	0	
Patients with AE leading to treatment interruption (%)	5.3	19.8	5.5	
Patients with AE leading to dose reduction (%)	5.7	7.1	0	
First subsequent therapy				
Abiraterone (%)	27	24.1	17	75.7
Enzalutamide (%)	23	3.4	0	12.1
Darolutamide (%)	‐	‐	33	0
Docetaxel (%)	23	17.1	50	9.1
Cabazitaxel (%)	0	1.1	0	0.6
Radium‐223 (%)	4	2.3	0	0
Others^a^ (%)	23	52	0	2.5
First subsequent therapy because of disease progression				
Abiraterone (%)	18.2	‐	25	‐
Enzalutamide (%)	0	‐	0	‐
Darolutamide (%)	0	‐	0	‐
Docetaxel (%)	54.5	‐	75	‐
Radium‐223 (%)	9.1	‐	0	‐
Others^a^ (%)	18.2	‐	0	‐
First subsequent therapy because AE				
Abiraterone (%)	33.3	‐	0	‐
Enzalutamide (%)	40	‐	0	‐
Darolutamide (%)	0	‐	100	‐
Docetaxel (%)	0	‐	0	‐
Radium‐223 (%)	0	‐	0	‐
Others^a^ (%)	26.7	‐	0	‐

*Note*: The numerators and denominators per variable adjusted according to the amount of valid data in this study are described in Table S3.

^a^ Includes: new clinical trials, androgen depletion therapy only, other types of therapy, other types of chemotherapies and other types of hormonal therapies.

### 3.4 Analyzed by subgroups

In the older age groups, a lower frequency of patients achieving PSA90 was observed. In the mHSPC group, PSA90 was achieved in 100% of patients <65 years, 94.2% of 65–74 years and 88.8% of >75 years. In the nmCRPC group, PSA90 was achieved in 100% of patients <65 years, 100% of 65–74 years and 25% of >75 years. In addition, the nadir PSA was higher in ≥75 years. When stratified by ECOG‐PS functional status, in the mHSPC group, we observed that the higher the ECOG‐PS score, the lower the frequency of patients achieving PSA90 or PSA ≤0.2 and the longer the time to achieve PSA endpoints. However, in the nmCRPC group, an adequate assessment could not be made due to the lack of data. When results were stratified by diagnostic imaging technique the results were inconclusive when analyzed by PSA endpoints. Subgroup analysis of primary endpoints of pivotal trials was not performed due to the low number of patients with disease progression. Table 4 in the supplementary appendix describes the data from the subgroup analyses.

## 4 DISCUSSION

Apalutamide is one of the most frequently used first‐line therapies for patients with high‐risk nmCRPC and mHSPC.^16^ Therefore, it is important to understand if the outcomes in real‐world patients reflect the efficacy data described in pivotal trials.^15^ In this real‐world study, an important and heterogeneous population representation was obtained, due to the diversity of patients collected from multiple medical disciplines and medical centres.

In our study, there are several important baseline demographic differences compared to the SPARTAN and TITAN trials. For instance, patients treated in our real‐world cohort were older, with higher ECOG PS (including grade 2), a higher proportion of high‐grade Gleason and higher median baseline PSA.^4^, ^5^ On the other hand, mHSPC patients in our study had a higher proportion of metachronous metastatic disease, and a higher proportion of low‐volume, low‐risk disease compared to the TITAN trial.^5^ The latter is due to the inclusion in our study of patients with lymph node‐only metastases, a patient population that was not included in TITAN trial, in whom the use of apalutamide is approved by the Spanish public health system.^17^

The nmCRPC population included in our series should be considered more advanced compared to SPARTAN, as analysis by disease stage showed that our patients had a higher proportion of PSADT ≤6 months compared to the SPARTAN trial.^4^ Of note, the Spanish public health system does not reimburse apalutamide for nmCRPC with PSADT >6 months.^18^

The important role of PSA kinetics in the follow‐up of apalutamide treatment in pivotal trials is well documented.^9^, ^10^ However, there are no studies that widely extrapolate these results to the real‐world clinical setting. Therefore, in our study, the times and end points of PSA response were analyzed, with a successful PSA response observed in all patients, and with more than half of the patients obtaining a PSA reduction greater than 90%. The median time to PSA response endpoints (PSA50, PSA90 and PSA ≤0.2) was <3 months. The median time to PSA nadir was 3 months in mHSPC and 6 months in nmCRPC. The median nadir PSA in mHSPC was 0.04, similar to 0.02 in the TITAN trial population and 0.5 in nmCRPC, higher than the 0.38 reported in the SPARTAN trial population.^9^, ^10^ This difference in the non‐metastatic population may be due to a higher median baseline PSA reported in our study.

Regarding safety, an overall lower percentage of AE was observed in our cohort compared to the pivotal trials. Indeed, this is not surprising as the evaluation and reporting of AE are more strict within a clinical trial and patients in real‐world practice may only refer to the most severe symptoms or these may be underreported if already managed by family doctors or other specialities.^19^, ^20^ Our results are similar to the lower frequency of AE found in a similar real‐world study by Hussain and colleagues.^21^

When analyzing the first therapy administered after apalutamide discontinuation, a predominance of chemotherapy use was observed when discontinuation was due to disease progression in comparison to patients in the pivotal trials who were treated equally with chemotherapy or a new hormonal agent (TITAN trial), or mostly with abiraterone acetate plus prednisone (SPARTAN trial).^4^, ^5^ One of the most probable reasons for that difference is the fact that abiraterone was provided cost‐free within the SPARTAN trial. In contrast, when the discontinuation was attributed to AEs, a second hormonal agent was preferred, allowing delayed initiation of chemotherapy similar to hormone‐based pivotal trials.^4^, ^5^, ^7^, ^8^

To our knowledge, this is the first extensive and detailed real‐world study including all patients treated with apalutamide either in the mHSPC and nmCRPC setting evaluating oncological outcomes, adverse events, and subsequent treatment lines at apalutamide discontinuation. In addition, a strength of our study is the fact that our cohort contains patients not included in the pivotal studies such as patients with lymph node metastases only, ECOG performance status of 2, or diagnosed with new‐generation imaging. The use of molecular imaging is increasing in the real‐world setting, thus having information on these patients is another strength of our study, as patients in the pivotal studies are underdiagnosed by using only conventional imaging.^6^, ^22^, ^23^, ^24^, ^25^

Although the results of our investigation are consistent with the successful therapy responses described by the pivotal studies, we also recognize that this is still a small population with short follow‐up. Consequently, this study is limited in the power of the conclusions drawn from the results analysed. We remark on the inherent limitations of real‐world studies based on medical records (treatment adherence by the patient, coding inaccuracies by medical staff, etc.).^12^, ^14^, ^15^ Therefore, because we did not have the necessary data to perform statistical tests, we could not assess the significance of the differences between our records and the pivotal studies. In addition, our study is a real‐world multi‐centre. Thus, the heterogeneity and lack of data were unavoidable, leading to variation in denominators when performing descriptive statistics.

In conclusion, although including an older and more comorbid patient population, the efficacy and safety profile of apalutamide was similar to that described in the pivotal trials. Subsequent therapies after apalutamide differ depending on the reason for the discontinuation, chemotherapy is more common after disease progression, and hormonal sequencing is more common after severe adverse events.

## AUTHOR CONTRIBUTIONS

**Julián Córdoba Sánchez:** Conceptualization (equal); data curation (equal); formal analysis (equal); investigation (equal); methodology (equal); project administration (equal); resources (equal); software (lead); validation (equal); visualization (lead); writing – original draft (lead); writing – review and editing (equal). **Natalia Picola:** Data curation (equal); writing – review and editing (equal). **Alejo Rodriguez‐Vida:** Data curation (equal); writing – review and editing (equal). **Marc Costa:** Data curation (equal); writing – review and editing (equal). **David Marmolejo Castañeda:** Data curation (equal); writing – review and editing (equal). **Meritxell Pérez Márquez:** Data curation (equal); writing – review and editing (equal). **Jesus Muñoz‐Rodriguez:** Data curation (equal); writing – review and editing (equal). **J. M. Gaya:** Data curation (equal); writing – review and editing (equal). **Alejandra Bravo:** Data curation (equal); writing – review and editing (equal). **Oscar Buisan:** Data curation (equal); writing – review and editing (equal). **Pol Servian:** Data curation (equal); writing – review and editing (equal). **Jose Francisco Suárez:** Data curation (equal); writing – review and editing (equal). **Mireia Musquera Felip:** Investigation (equal); writing – review and editing (equal). **Maria Jose Ribal Caparrós:** Funding acquisition (lead); investigation (lead); writing – review and editing (lead). **Antonio Alcaraz Asensio:** Funding acquisition (lead); investigation (lead); writing – review and editing (lead). **Antoni Vilaseca:** Conceptualization (lead); data curation (lead); formal analysis (lead); funding acquisition (lead); investigation (lead); methodology (lead); project administration (lead); resources (lead); supervision (lead); validation (lead); visualization (lead); writing – review and editing (lead).

## CONFLICT OF INTEREST STATEMENT

This study did not receive funding. Julián Córdoba and Meritxell Pérez have received sponsorship from Janssen for medical congresses and symposiums. Alejo Rodriguez‐Vida, Jesús Muñoz Rodriguez, Antonio Alcaraz and Antoni Vilaseca have received honoraria from Janssen for advisory board meetings, symposiums and travel expenses. The other authors declare no conflict of interest. Approval of the research protocol by an Institutional Reviewer Board: HCB/2019/0919.

## Supporting information


Table S1.
Table S2.
Table S3.

## Data Availability

The data that support the findings of this study are available from the corresponding author upon reasonable request.
