# Supplementary material for: Apalutamide for prostate cancer: Multicentre and multidisciplinary real‐world study of 227 patients
Source: Cancer Med. 2023 Dec 8;12(24):21969–77. doi: 10.1002/cam4.6769 (PMC10757138; doi:10.1002/cam4.6769)
Supplement: Supplementary file 1 — Table S1. Table S2. Table S3. [file CAM4-12-21969-s001.docx]

**SUPPLEMENTARY APPENDIX**

In this study, at the time of the analysis of results, the denominator was adjusted according to the availability of valid data for each variable studied. Supplementary tables 1, 2 and 3 detail the numbers of validated data and denominators for each variable analysed in tables 1, 2 and 3 of the main manuscript.

| **Supplementary Table 1. N valid data from Characteristics of the Patients and Oncological Outcomes.** | | |
| --- | --- | --- |
|  | **mHSPC** | **nmCRPC** |
| Total of patients, *n* | 209 | 18 |
| Median Follow-up, *n valid data* | 207 | 18 |
| Median Age, *n valid data* | 209 | 18 |
| ECOG PS, *n - denominator* | 157 | 11 |
| 0, *n* | 92 | 8 |
| 1, *n* | 50 | 1 |
| 2, *n* | 15 | 2 |
| Diagnostic Technique, *n - denominator* | 205 | 18 |
| Conventional Imaging, *n* | 118 | 13 |
| New Generation Imaging, *n* | 87 | 5 |
| Gleason score, n - denominator | 202 | 18 |
| <7, *n* | 1 | 1 |
| 7, *n* | 64 | 6 |
| >7, *n* | 137 | 11 |
| Metastatic Disease, *n - denominator* | 203 | - |
| Synchronous, *n* | 108 | - |
| Metachronous, *n* | 95 | - |
| Metastatic Stage, *n - denominator* | 209 | - |
| M1a, *n* | 45 | - |
| M1b-c, *n* | 164 | - |
| Disease Volume, *n - denominator* | 209 | - |
| Low, *n* | 136 | - |
| High, *n* | 73 | - |
| Disease Risk, *n - denominator* | 209 | - |
| Low, *n* | 127 | - |
| High, n | 82 | - |
| PSA doubling time, *n - denominator* | - | 16 |
| ≤6 Mo, *n* | - | 13 |
| >6 Mo, *n* | - | 3 |
| PSA baseline, *n valid data* | 209 | 18 |
| PSA 1mo, *n valid data* | 179 | 18 |
| PSA 3mo, *n valid data* | 187 | 15 |
| PSA 6mo, *n valid data* | 150 | 10 |
| PSA 12mo, *n valid data* | 90 | 8 |
| The other variables corresponding to PSA kinetics were calculated based on the PSA valid data for each Time Point. | | |

| **Supplementary Table 2. *N* valid dates from Adverse Events (AE).** | | |
| --- | --- | --- |
|  | **mHSPC** | **nmCRPC** |
| Patients with AE, *n valid dates.* | 84 | 7 |
| Any AE and All grades, *n – denominator.* | 94 | 8 |
| Any AE and grade ≥3, *n valid dates.* | 24 | 3 |
| Any AE leading to death, *n valid dates.* | 0 | 0 |
| Time to AE – *days,* *n valid dates.* | 91 | 8 |
| Time to AE resolution – *days, n valid dates.* | 53 | 3 |
| Most Frequent Adverse Events, *n* valid dates | | |
| Rash - All grades, *n valid dates.* | 33 | 3 |
| Grade ≥3, *n valid dates.* | 12 | 2 |
| Asthenia or Fatigue - All grades, *n valid dates.* | 38 | 1 |
| Grade ≥3, *n valid dates.* | 6 | 1 |
| Arthralgia - All grades, *n valid dates.* | 5 | 1 |
| Grade ≥3, *n valid dates.* | 2 | 0 |
| Hypertension - All grades, *n valid dates.* | 5 | 1 |
| Grade ≥3, *n valid dates.* | 3 | 0 |
| Weight alteration - All grades, *n valid dates.* | 2 | 0 |
| Grade ≥3, *n valid dates.* | 1 | 0 |
| Diarrhea or Constipation - All grades, *n valid dates.* | 3 | 2 |
| Grade ≥3, *n valid dates.* | 0 | 0 |
| Hypothyroidism - All grades | 3 | 0 |
| Grade ≥3, *n valid dates.* | 0 | 0 |
| Blood alkaline phosphatase increased - All grades, *n valid dates.* | 1 | 0 |
| Grade ≥3, *n valid dates.* | 0 | 0 |

| **Supplementary Table 3. *N* valid date from Modification of Treatment and Subsequent Therapy.** | | |
| --- | --- | --- |
|  | **mHSPC** | **nmCRPC** |
| Patients who discontinued apalutamide for any reason, *n valid date.* | 29 | 8 |
| Patients who discontinued apalutamide because of disease progression, *n valid date.* | 15 | 6 |
| Time to discontinued apalutamide because of disease progression, *n* valid date. | 15 | 6 |
| Patients with AE leading to discontinuation of apalutamide, *n valid date.* | 12 | 2 |
| Time to discontinuation of apalutamide because AE, *n valid date.* | 11 | 2 |
| Deaths from any cause, *n valid date.* | 2 | 0 |
| Patients with AE leading to treatment interruption, *n valid date.* | 10 | 1 |
| Patients with AE leading to dose reduction, *n valid date.* | 13 | 0 |
| First subsequent therapy, *n – denominator.* | 26 | 6 |
| Abiraterone, *n valid date.* | 7 | 1 |
| Enzalutamide, *n valid date.* | 6 | 0 |
| Darolutamide, *n valid date.* | - | 2 |
| Docetaxel, *n valid date.* | 6 | 3 |
| Radium-223, *n valid date.* | 1 | 0 |
| *Others, *n valid date.* | 6 | 0 |
| Subgroups by cause of discontinuation | | |
| First subsequent therapy because of Disease Progression, *n – denominator.* | 11 | 4 |
| Abiraterone, *n valid date.* | 2 | 1 |
| Enzalutamide, *n valid date.* | 0 | 0 |
| Darolutamide, *n valid date.* | - | 0 |
| Docetaxel, *n valid date.* | 6 | 3 |
| Radium-223, *n valid date.* | 1 | 0 |
| *Others, *n valid date.* | 2 | 0 |
| First subsequent therapy because of AE, *n – denominator.* | 15 | 2 |
| Abiraterone, *n valid date.* | 5 | 0 |
| Enzalutamide, *n valid date.* | 6 | 0 |
| Darolutamide, *n valid date.* | - | 2 |
| Docetaxel, *n valid date.* | 0 | 0 |
| Radium-223, *n valid date.* | 0 | 0 |
| *Others, *n valid date.* | 4 | 0 |
| * Includes: new clinical trials, androgen depletion therapy only, other types of therapies, other types of chemotherapies and other types of hormonal therapies. | | |

| **Table 4. Analysis by Subgroups** | | | | | | |
| --- | --- | --- | --- | --- | --- | --- |
|  | **Analysis by Age Range** | | | | | |
|  | **mHSPC** | | | **nmCRPC** | | |
|  | <65 | 65-74 | ≥75 | <65 | 65-74 | ≥75 |
| Total of patients, *n valid data* | 25 | 72 | 112 | 2 | 4 | 12 |
| PSA baseline (ng/ml). median | 19.1 | 6.9 | 17.0 | 17.4 | 2.84 | 13.3 |
| (IQR) | 63.4 | 30.3 | 48.8 | 15.2 | 4.57 | 20.8 |
| Patients achieving PSA response in any time (%) |  |  |  |  |  |  |
| Any decrease | 100 | 100 | 100 | 100 | 100 | 100 |
| PSA 50 | 100 | 100 | 100 | 100 | 100 | 83.3 |
| PSA 90 | 100 | 94.2 | 88.8 | 100 | 100 | 25 |
| PSA ≤ 0.2 | 88 | 78.2 | 63.8 | 50 | 75 | 25 |
| Time to achieve PSA response-mo. |  |  |  |  |  |  |
| Any decrease, median | 1.0 | 1.0 | 1.0 | 1.0 | 1.0 | 1.0 |
| (IQR) | 0.0 | 0.0 | 0.0 | 0.0 | 0.0 | 0.0 |
| PSA 50, median | 1.0 | 1.0 | 1.0 | 1.0 | 1.0 | 1.0 |
| (IQR) | 0.0 | 0.0 | 0.0 | 0.0 | 0.0 | 2.0 |
| PSA 90, median | 1.0 | 1.0 | 1.0 | 1.0 | 3.0 | 1.0 |
| (IQR) | 2.0 | 2.0 | 2.0 | 0.0 | 1.3 | 1.0 |
| PSA ≤ 0.2, median | 3.0 | 3.0 | 3.0 | 1.0 | 3.0 | 3.0 |
| (IQR) | 2.0 | 2.0 | 2.0 | 0.0 | 2.5 | 2.5 |
| PSA nadir (ng/ml). Median | 0.04 | 0.04 | 0.06 | 0.09 | 0.03 | 3.5 |
| (IQR) | 0.01 | 0.05 | 0.27 | 0.0 | 0.0 | 20.8 |
| Time to PSA nadir. median – mo. | 3.0 | 3.0 | 6.0 | 3.0 | 9.0 | 6.0 |
| (IQR) | 7.0 | 3.0 | 3.0 | 0.0 | 3.0 | 9.0 |
|  | **Analysis by ECOG-PS** | | | | | |
|  | **mHSPC** | | | **nmCRPC** | | |
|  | 0 | 1 | 2 | 0 | 1 | 2 |
| Total of patients. *n valid data* | 89 | 49 | 15 | 8 | 1 | 2 |
| PSA baseline (ng/ml). median | 9.37 | 33.2 | 123.0 | 13.4 | 48.0 | 48.2 |
| (IQR) | 30.5 | 139.0 | 396.0 | 24.2 | 0.0 | 35.1 |
| Patients achieving PSA response in any time (%) |  |  |  |  |  |  |
| Any decrease | 100 | 100 | 100 | 100 | 100 | 100 |
| PSA 50 | 98.8 | 100 | 93.3 | 87.5 | 100 | 100 |
| PSA 90 | 93.2 | 97.9 | 80 | 62.5 | 0 | 0 |
| PSA ≤ 0.2 | 79.7 | 75.5 | 46.6 | 37.5 | 0 | 0 |
| Time to achieve PSA response-mo. |  |  |  |  |  |  |
| Any decrease, median | 1.0 | 1.0 | 1.0 | 1 | 3 | 1 |
| (IQR) | 0.0 | 0.0 | 1.0 | 0 | 0 | 0 |
| PSA 50, median | 1.0 | 1.0 | 1.0 | 1 | 3 | 1 |
| (IQR) | 0.0 | 0.0 | 1.5 | 1 | 0 | 0 |
| PSA 90, median | 1.0 | 2.0 | 2.0 | 1 | 0 | 0 |
| (IQR) | 2.0 | 2.0 | 2.0 | 2 | 0 | 0 |
| PSA ≤ 0.2, median | 3.0 | 3.0 | 4.5 | 3 | 0 | 0 |
| (IQR) | 2.0 | 5.0 | 3.0 | 1 | 0 | 0 |
| PSA nadir (ng/ml). Median | 0.04 | 0.05 | 0.25 | 0.5 | - | 14.5 |
| (IQR) | 0.05 | 0.07 | 2.37 | 6.65 | - | 11 |
| Time to PSA nadir. median – mo. | 3.0 | 6.0 | 4.5 | 6 | - | 3 |
| (IQR) | 3.0 | 3.0 | 3.0 | 6.75 | - | 0 |
|  | **Analysis by Diagnostic Imaging Technique** | | | | | |
|  | **mHSPC** | | | **nmCRPC** | | |
|  | CI | NGI | | CI | NGI | |
| Total of patients. n *valid data* | 118 | 87 | | 13 | 5 | |
| PSA baseline (ng/ml). median | 33.0 | 3.72 | | 11.4 | 18.7 | |
| (IQR) | 112.0 | 11.2 | | 22.1 | 17.7 | |
| Patients achieving PSA response in any time (%) |  |  | |  |  | |
| Any decrease | 100 | 100 | | 100 | 100 | |
| PSA 50 | 97.4 | 98.7 | | 92.3 | 80 | |
| PSA 90 | 91.3 | 93.9 | | 46.1 | 60 | |
| PSA ≤ 0.2 | 66.3 | 79.5 | | 46.1 | 20 | |
| Time to achieve PSA response-mo. |  |  | |  |  | |
| Any decrease, median | 1.0 | 1.0 | | 1.0 | 1.0 | |
| (IQR) | 0.0 | 0.0 | | 0.0 | 0.0 | |
| PSA 50, median | 1.0 | 1.0 | | 1.0 | 1.0 | |
| (IQR) | 0.0 | 0.0 | | 0.0 | 0.0 | |
| PSA 90, median | 1.0 | 1.0 | | 1.0 | 3.0 | |
| (IQR) | 2.0 | 2.0 | | 1.5 | 2.5 | |
| PSA ≤ 0.2, median | 3.0 | 3.0 | | 2.0 | 6.0 | |
| (IQR) | 5.0 | 2.0 | | 2.0 | 0.0 | |
| PSA nadir (ng/ml). Median | 0.06 | 0.04 | | 0.5 | 10.5 | |
| (IQR) | 0.21 | 0.03 | | 7.3 | 10.5 | |
| Time to PSA nadir. median – mo. | 6.0 | 3.0 | | 6.0 | 7.5 | |
| (IQR) | 3.0 | 3.0 | | 7.5 | 4.5 | |
| The numerators and denominators per variable adjusted according to the amount of valid data in this study. | | | | | | |
